# Supplementary material for: Content-rich biological network constructed by mining PubMed abstracts
Source: BMC Bioinformatics. 2004 Oct 8;5:147. doi: 10.1186/1471-2105-5-147 (PMC528731; doi:10.1186/1471-2105-5-147)
Supplement: Additional File 2 — The original results of the above study (non-essential files are deleted to keep the file size under the limit set by BMC bioinformatics). [file 1471-2105-5-147-S2.bz2 › chilibotAdditionalFile2/dip20/155ID8822197E554/html/RB1_CDK6.html]

 


 **RB1** and **CDK6** 
  
Found 151 abstracts in PubMed, retrieved 20.  
 

 What does Google say? 
 PDF only 
| .edu only 

---

**Interactive relationship** (e.g. stimulation, inhibition, etc)

**Inhibitory relationship**- At the molecular level, the tetramer inhibited cyclin dependent kinase CDK 6  [ **CDK6** ]  expression and activity, induced an increase in the level of p27 kip1, and reduced the level of retinoblastoma  [ **RB1** ]  protein expression.  Ref: 12874002 Cancer ResCancer Res,
**Neutral relationship**- In human T lymphocytes  **cdk6**  has been shown to be the initial retinoblastoma  [ **RB1** ]  protein kinase detectable at mid G1.  Ref: 11804329 Mol Cells, 2001
- The phosphorylation of retinoblastoma  [ **RB1** ]  protein from T cells was detected very early in G1 and was associated mainly with  **cdk6**  cyclin D2 complexes which accounted for a minor portion of the total cellular  **cdk6**  contained in the cytoplasmic fraction of T cells and mostly in the catalytically inactive form.  Ref: 11804329 Mol Cells, 2001
- D type cyclins bind to and activate the cyclin dependent kinases Cdk4 and  **Cdk6** , which in turn phosphorylate their downstream target, the retinoblastoma  [ **RB1** ]  protein Rb.  Ref: 12459251 Gene, 2002

**Non-interactive relationship** (e.g. studied together, co-existance, homology, etc.)

- In HepG2 cells, this inhibition was associated with an arrest of the cell cycle in G G, increased cellular levels of p21, decreased levels of the hyperphosphorylated form of the retinoblastoma  [ **RB1** ]  protein, and decreased levels of cyclin D1, but no significant changes were seen in the levels of the p16, p27, cyclin dependent kinase 4, cyclin dependent kinase 6  [ **CDK6** ] , glycogen synthase kinase 3beta, or beta catenin proteins.  Ref: 12124333 Cancer Res, 2002
- Components upstream of the retinoblastoma  [ **RB1** ]  tumour suppressor pRB, namely the INK4 family of cyclin dependent kinase CDK inhibitors, the D type cyclins, their partner kinases CDK4  **CDK6** , and pRB as their critical substrate, are differentially targeted in diverse types of cancer.  Ref: 12754735 J Pathol< MedlineTA>J Pathol, 2002
- p16 regulates the G 1 S cell cycle transition by inhibiting the cyclin D cyclin dependent kinase CDK 4  **CDK6**  mediated phosphorylation of retinoblastoma  [ **RB1** ]  protein pRb.  Ref: 11705866 Clin Cancer Res, 2001
- Abrogation of mitogenic and anti mitogenic response regulatory proteins, such as the retinoblastoma  [ **RB1** ]  tumor suppressor protein, cyclin D1, cyclin dependent kinase 6  [ **CDK6** ] , and CDK inhibitors, p27, and p16, occur frequently in human oral cancers.  Ref: 12097237 Crit Rev Oral Biol Med, 2002
